# Islamic virtues and end‐of‐life decisions in clinical practice: A commentary on Mehrunisha Suleman, ‘The Balancing of Virtues—Muslim Perspectives on End of Life Care: Empirical research analysing the perspectives of service users and providers’

**DOI:** 10.1111/bioe.13118

**Published:** 2022-12-04

**Authors:** Justin Oakley

**Affiliations:** ^1^ Monash Bioethics Centre, School of Philosophical, Historical and International Studies Monash University Melbourne Victoria Australia

In her detailed study of Muslim perspectives on end‐of‐life care, Mehrunisha Suleman provides an illuminating discussion of reflections by both Muslim and non‐Muslim practitioners and patients about death and dying.^1^ Suleman's findings and her analyses of these are very insightful, while they also raise some challenges for doctors’ roles in end‐of‐life care.

In Islamic ethics, as Suleman explains, the Qur'an and the Hadith provide the foundations for understanding both *adab* (virtue) and *aqhlaq* (proper conduct). For example, the frequent mention in the Qur'an and in the Hadith of *sabr* (patience) in adversity ‘signifies life as being a test, replete with adversity and challenges’, just as ‘The Prophet Muhammad's life is replete with death, loss and bereavement’.^2^ As Elizabeth Bucar explains, ‘hadith are brief written reports of things the Prophet Muhammad said or did. They are used to help “fill in” where Qur'anic revelation is silent’.^3^

Suleman highlights how Muslim patients see a good death and dying as a deeply spiritual process, which needs to harmonise with their faith commitments: ‘Patients and families describe faith commitments that not only include fulfilling ritualistic requirements around death… but also [as involving] an overarching spiritual process of approaching, understanding and experiencing life to its absolute end’.^4^ It is interesting to see the centrality for Muslim patients of the virtues of hope and acceptance in this process. Suleman explains that ‘the data from this study show that for Muslim patients and families the virtues of hope and acceptance not only coexist but are integral to their meaning making around death and dying, inform what they consider to be a “good death” and govern the care they seek and expect from the healthcare professionals they encounter’.^5^ The virtue of hope in this context seems to involve hoping for one's life to continue longer, while the virtue of acceptance seems to include an understanding of and commitment to ‘one's imminent departure’.^6^

On the face of it, these attitudes appear to be in tension with one another. However, Suleman clarifies how these attitudes can consistently coexist in the dying, insofar as they both involve *taqwah*, that is, faith and belief in (and consciousness of) God: ‘the singular psychological state of *taqwah* enables the coexisting of two or even multiple virtues and … there may not necessarily be a balancing or trade‐off of different virtues, rather that at different times, different aspects of *taqwah* are manifest’.^7^ Suleman describes how Muslim chaplains draw on such understandings in considering how to care for patients and families and in ministering to the dying and to grieving families.^8^

One of the Muslim palliative care doctor interviewees mentions patients’ ‘faith that life and death are in Allah's hands. And so you have nothing to say about it. So if there is something you can do, you should do it’.^9^ This doctor seems to be saying here that it is not for *him* (or for the patient themselves) to decide that a person dies, or when they die. In saying ‘if there is something you can do, you should do it’, is this doctor expressing the view that Muslim doctors should keep dying patients alive, whether or not this is in the patient's overall best interests? If so, such a view might seem to condone the doctor's personal moral and religious beliefs overriding the proper boundaries of their professional role and social remit, which seems to be morally questionable. Note that in questioning this, I am not suggesting that a doctor can never justifiably conscientiously object on religious grounds to providing a medical procedure. Indeed, I believe that it can be justifiable under certain conditions for doctors to conscientiously refuse on religious grounds to provide a medical procedure—particularly when the procedure in question is a peripheral rather than a core part of medical practice. (One example where a doctor conscientiously refusing on religious grounds to provide a core medical service seems unjustifiable might be a Jain doctor refusing to prescribe a needed antibiotic to a patient on the grounds that the doctor believes that even bacterial life has intrinsic value.)

Rather, I am making a more general point here. Any theory of medical ethics must acknowledge that, among a doctor's personal moral beliefs, there will be *some* that should not impede their fulfilling their professional roles as doctors. At some level, there needs to be a distinction between a doctor refusing to provide a particular service to a patient because the doctor believes that this service is contrary to the proper goals of medicine, and a doctor refusing to provide a particular service to a patient because the doctor believes that providing this service would violate the doctor's deep moral convictions. For example, some doctors in the Australian state of Victoria are opposed to being involved in providing voluntary assisted dying because they regard such a practice as contrary to the proper goals of medicine, and so as contrary to the requirements of their role. However, not all of those doctors are *personally* morally opposed (e.g., on religious grounds) to voluntary assisted dying. In the first type of refusal case, doctors can be understood as appealing to *professional integrity* as their grounds for refusing to provide the service in question (even if those doctors’ belief that such a practice is contrary to the proper goals of medicine can be questioned^10^), whereas in the second sort of refusal case, doctors can be understood as appealing to *personal integrity* as their grounds for refusing to provide the service in question. In the former case, the doctor is giving a *role*‐relative reason for refusing to provide the service—that is, they are claiming that ‘No good doctor would provide this procedure’—whereas in the latter case, the doctor is giving an *agent*‐relative reason for refusing to provide the service—that is, they are saying that ‘I cannot in good conscience provide this procedure’—without necessarily saying that no good doctor would provide the service (as the doctor might concede that there is reasonable disagreement about whether such a practice is consistent with the proper goals of medicine).^11^ And a doctor who advises that any patients who can be kept alive, *should* be kept alive, could be regarded as recommending what might amount to complicity in a failure of *professional* virtue, in circumstances where keeping the patient alive is not in the patient's best interests.

In response, it might be argued that whenever a doctor (whether Muslim or non‐Muslim) extends the life of a dying patient who is Muslim, the doctor *would* actually be acting in the best overall interests of that patient, insofar as this is consistent with a Muslim patient's faith commitments.

However, does the social remit for doctors extend to serving patients’ religious interests? A key question here is whether it is plausibly regarded as part of a doctor's professional role to help patients act virtuously, or to become better, more virtuous individuals—say, in circumstances where this does not seem to serve the health of patients or the community. For example, while it may be in a patient's *overall* best interests to develop various civic virtues through becoming more politically engaged citizens, would this justify a doctor encouraging a patient to develop such virtues, in circumstances where there are no foreseeable health gains for the patient or the community in their doing so?

Let us now return to the virtues of hope and acceptance in dying patients. Suleman presents some interesting reflections from a non‐Muslim palliative care nurse in this context, who ‘spoke of the difficulty in being able to understand and accommodate for patients’ and families’ commitment in hope—a hope for a cure, a miracle—that is reliant on God. She explained that although hope can be integral to people's coping mechanism, she was also committed to ensuring they had had the opportunity to prepare for their end of life’.^12^ Suleman explains that this nurse—and ‘many other healthcare professionals interviewed during the study’^13^—found it challenging to understand ‘the beliefs and commitments of Muslim patients and families based on the virtue of hope… Their steadfastness and hope may be perceived by healthcare professionals as a coping mechanism or even a manifestation of denial’.^14^ Suleman argues strongly that a key lesson from this is that non‐Muslim doctors need to better understand that both hope and acceptance can coherently coexist, as a request from a Muslim patient (or their family) for the provision of life‐extending interventions can be understood as helping the patient to maintain the virtue of hope, and need not indicate that the dying patient is in denial, or has failed to accept that they are (most likely) about to die.

This is a very important recommendation from the study. I wondered whether it might be helpful to highlight how hope and acceptance have different objects here, and can manifest at different times while a patient is dying. Hope can coherently coexist with acceptance in other contexts. For example, one can accept that, as one approaches Moscow on a Trans‐Siberian rail journey, one's week‐long journey is about to come to an end, while also hoping (even without being a rail enthusiast) that Moscow might be that little bit further off (perhaps because signalling delays might extend one's journey with one's delightful compartment companions for a little longer). So, perhaps illustrating the coexistence of hope and acceptance in other contexts might help non‐Muslim doctors to understand how these can both be present, and need to be properly acknowledged, in end‐of‐life care.

## CONFLICT OF INTEREST

The author declares no conflict of interest.

## Footnotes

1 Suleman, M. (2023). The Balancing of Virtues—Muslim Perspectives on End of Life Care: Empirical research analysing the perspectives of service users and providers. *Bioethics*, *37*, 57–68. https://doi.org/10.1111/bioe.13109.

2 Ibid: 8.

3 Bucar, E. M. (2018). Islamic virtue ethics. In N. E. Snow (Ed.), *The Oxford handbook of virtue* (pp. 206–223). Oxford University Press, p. 207.

4 Suleman, op. cit. note 1, p. 7.

5 Ibid: 10.

6 Ibid: 9.

7 Ibid: 11.

8 Ibid: 7.

9 Ibid: 10.

10 The assumption that providing voluntary assisting dying to a patient at their autonomous request can never be in the patient's interests and so could never be a healing act is highly questionable. Indeed, the broad concept of healing as ‘making whole’, which is often employed by those (such as Leon Kass) who make such an assumption, can enable a good death to be understood as the completion or making whole of the life of a patient, in certain circumstances. See Oakley, J., & Cocking, D. (2001). *Virtue ethics and professional roles* (pp. 83–84). Cambridge University Press; Kass, L. (1989). Neither for love nor money: Why doctors must not kill. *The Public Interest*, *94*, 25–46.

11 See Oakley, J. (2013). Virtue ethics and bioethics. In D. Russell (Ed.), *Cambridge companion to virtue ethics* (pp. 197–220). Cambridge University Press, p. 207.

12 Suleman, op cit. note 1, p. 9.

13 Ibid.

14 Ibid.

